# TECHNOLOGY-BASED HOME MONITORING TO ASSESS AND IMPROVE THE HEALTH AND WELL-BEING OF OLDER ADULTS

**DOI:** 10.1093/geroni/igz038.1354

**Published:** 2019-11-08

**Authors:** Walter R Boot

**Affiliations:** Florida State University, Tallahassee, Florida, United States

## Abstract

Technology, including in-home sensors, monitoring systems, and wearables, holds great promise with respect to being able to help manage chronic conditions, improve older adults’ health, support their safety, prolong independence, and collect data for healthcare and research purposes. This symposium will introduce novel ways in which these systems are being deployed, and also important challenges to their implementation that could decrease their potential benefits. This technology will only be successful if it addresses key issues of adherence and privacy, and considers users’ preferences. The first talk in this session will explore the novel use of GPS-based wearable sensors to capture the concept of life-space mobility, a measure critically related to quality of life and independence. The next talk focuses on a mobile health cardiovascular system and smartphone application, and finds that such systems can successfully transmit meaningful mHealth data while maintaining reasonable protocol adherence. Next, issues of user preference are discussed, including the types of data older adults and their children are willing to share, and the tradeoffs between values of privacy and independence. The session will conclude with a talk on older adults’ attitudes toward privacy related to in-home monitoring, and individual difference predictors of privacy concerns. In sum, this session will provide an overview of the potential and pitfalls related to in-home sensors and monitoring systems for older adults, their formal and informal caregivers, and aging researchers.

